# Ulinastatin Ameliorates IL-1*β*-Induced Cell Dysfunction in Human Nucleus Pulposus Cells via Nrf2/NF-*κ*B Pathway

**DOI:** 10.1155/2021/5558687

**Published:** 2021-04-21

**Authors:** Xi Luo, Le Huan, Feng Lin, Fanqi Kong, Xiaofei Sun, Fudong Li, Jian Zhu, Jingchuan Sun, Ximing Xu, Kaiqiang Sun, Liwei Duan, Jiangang Shi

**Affiliations:** ^1^Department of Orthopedic Surgery, Changzheng Hospital, Navy Medical University (Second Military Medical University), No. 415 Fengyang Road, Shanghai 200003, China; ^2^Department of Emergency and Critical Care Medicine, Changzheng Hospital, Naval Medical University (Second Military Medical University), Shanghai 200433, China

## Abstract

Low back pain (LBP) has been a wide public health concern worldwide. Among the pathogenic factors, intervertebral disc degeneration (IDD) has been one of the primary contributors to LBP. IDD correlates closely with inflammatory response and oxidative stress, involving a variety of inflammation-related cytokines, such as interleukin 1 beta (IL-1*β*), which could result in local inflammatory environment. Ulinastatin (UTI) is a kind of acidic protein extracted from human urine, which inhibits the release of tumor necrosis factor alpha (TNF-*α*) and other inflammatory factors to protect organs from inflammatory damage. However, whether this protective effect of UTI on human nucleus pulposus (NP) exists, and how UTI affects the biological behaviors of human NP cells during IDD remain elusive. In this current study, we revealed that UTI could improve the viability of NP cells and promote the proliferation of NP cells. Additionally, UTI could protect human NP cells via ameliorating IL-1*β*-induced apoptosis, inflammatory response, oxidative stress, and extracellular matrix (ECM) degradation. Molecular mechanism analysis suggested that the protective effect from UTI on IL-1*β*-treated NP cells were through activating nuclear factor- (erythroid-derived 2-) like 2 (Nrf2)/heme oxygenase-1 (HO-1) signaling pathway and the suppression of NF-*κ*B signaling pathway. Therefore, UTI may be a promising therapeutic medicine to ameliorate IDD.

## 1. Introduction

Low back pain (LBP) has ranked as one of the major public concerns worldwide, with approximately 80% of the populations experiencing LBP in their lifetime [1]. Among various pathogenic contributors, intervertebral disc degeneration (IDD) has the mostly focused and studied [2].

Automatically, the intervertebral disc includes three main structures: the inner nucleus pulposus (NP), circumferential fibrocartilaginous annulus fibrosus (AF), and cartilaginous endplates (EP) that cap the NP and AF [3]. Under healthy condition, NP tissue is mainly composed of NP cells and extracellular matrix (ECM) (collagen type II and aggrecan) [4]. Plentiful studies have previously suggested that IDD is a pathological process which is closely related to inflammatory responses and oxidative stress, and involves a variety of inflammation mediators such as interleukin 1 beta (IL-1*β*) and tumor necrosis factor alpha (TNF-*α*) [5]. Furthermore, these inflammation cytokines will trigger the generation of a series of inflammatory factors, such as nitric oxide (NO) and prostaglandin E2 (PGE2), which can promote cell apoptosis, excessive inflammation reactions, and the generation of reactive oxygen species (ROS). Taken together, IDD will be initiated under the conditions above. Subsequently, these pathogenic changes may compromise the integrity of the intervertebral disc, cause abnormal distribution of mechanical load imposed on the spine, and consequently destroy the mechanical properties of the spine. At molecular level, nuclear tor-erythroid 2-related factor-2 (Nrf2) has been reported to process the role of antioxidant and anti-inflammation in many degeneration-related diseases [6]. Previous studies have demonstrated that activated nuclear factor- (erythroid-derived 2-) like 2 (Nrf2)/heme oxygenase-1 (HO-1) signaling pathway showed inhibitory effect on NF-*κ*B in NP cells. Tang et al. have also suggested that the expression of Nrf2 elevated in human degenerative NP tissue and that knocking down Nrf2 aggravated IDD [7]. Therefore, agents targeting at reversing or enhancing the expression of Nrf2 may be beneficial in the treatment of IDD.

Ulinastatin (UTI) is a kind of acidic protein extracted from human urine, which is a highly effective protease inhibitor with the function of stabilizing cell membrane and lysosomal membrane [8]. In addition, UTI can inhibit the release of tumor TNF-*α* and other inflammatory factors, prevent the interaction between inflammatory mediators and leukocytes, and depress aggregation and activation of leukocyte. UTI is clinically used to inhibit systemic inflammatory reactions such as pancreatitis and acute circulatory failure, presenting a wide range of pharmacological effects and significant protective effects on the heart, lung, kidney, brain, and other organs [9]. Hua et al. have studied the effect of UTI on degenerated NP cells of rabbits and found that UTI could inhibit the expression of MMP-2, MMP-3, and iNOS [10]. However, whether this protective effect of UTI on human NP remains equally valid and how UTI affects the biological behaviors of human NP cells remain elusive.

The objective of the present study is to examine the biological effects of UTI on human NP cells and the underlying molecular mechanisms. The evidence provided by this study will be of great significance for the clinical application of UTI in the treatment of IDD.

## 2. Methods and Materials

### 2.1. Acquisition and Patient Samples

The experiment was approved by the ethics committee from Changzheng Hospital, and the signed informed consent was acquired from all patients. The NP cells were collected from 12 patients (male 5, female 7, mean age 45.00 ± 9.07 years) from January 2020 to January 2021. All patients accepted preoperative MRI of the lumbar spine, and the Pfirrmann grading system was used to assess the condition of the affected disc. According to Pfirrmann grade, patients were divided into nondegeneration group (Grade II) and degeneration group (Grade IV). The nondegeneration group consisted of 6 patients with idiopathic scoliosis or lumbar trauma who required lumbar surgery, and all 6 patients in degeneration group were treated due to IDD and associated compression of nerve roots at lumbar spine.

### 2.2. Human NP Cells Isolation, Culture, and Identification

This method has been reported previously [11, 12]. After separated from patients intraoperatively, NP tissue was immediately stored in 0.9% sodium chloride solution and then transported to super clean bench. Following being washed twice using aseptic PBS, NP tissue was isolated with Trypsin-EDTA (0.25%, Gibco, Invitrogen) for 20-30 minutes and collagenase type II (0.2%, Invitrogen, Carlsbad, CA, USA) for another 3-4 h at 37°C combined with 0.1% of fetal bovine serum (FBS, Gibco; Thermo Fisher Scientific, Inc.) and 1% of a penicillin-streptomycin mix (Gibco; Thermo Fisher Scientific, Inc.). After isolation, NP cells were resuspended in DMEM/F12 with 20% of FBS and 1% penicillin-streptomycin solution and cultured at 37°C. Frequently, NP cells would move out of the fragment tissues after five days, which we could passage 0. When confluent to about 80%-90%, the cells were digested by Trypsin-EDTA for 2 minutes at 37°C. Next, cells were resuspended and replanted into 25 cm^3^ culture flasks with a proper density. Due to no obvious morphological changes of NP cells between initial cells (passage 1, P1) and following passage cells (P4), the low-passage (<4) cells were used for the following experiments. The phenotype of NP cells was validated by identifying the expression of type II collagen and aggrecan (Supplementary file 1).

### 2.3. Cell Viability Assay

In this section, cell Counting Kit-8 (CCK-8, Dojindo, Japan) was used. Briefly, the human NP cells were seeded in a 96-well plate with a density of 4 − 5 × 10^3^ cells/well. After cell adhesion of 24 hours, cells were stimulated by various concentrations of UTI (0-5000 U/mL) were for 24 h at 37°C. Next, 10 *μ*L CCK-8 solution combined with 100 *μ*L of DF-12 was added into all tested well, and then, the cells were incubated at the temperature of 37°C for 1 h. The absorbance was measured (450 nm) on an absorbance microplate reader (Bio-Tek, USA).

### 2.4. Real Time Quantitative PCR (qRT-PCR)

Total RNA was isolated from the human NP cells seeded in six-well plate with RNA extraction kit, and the concentration of mRNA was frequently 100 ng/*μ*L (Magen, Inc. Guangzhou, China). Then, cDNA was reversed using HiScript ® III RT SuperMix for qPCR Kit (R323-01, Vazyme, Nanjing, China), frequently with 2 *μ*L 5x III RT SuperMix, 1-2 *μ*L mRNA solution, and 6-7 *μ*L RNase-free ddH_2_O. The expression at mRNA levels of anabolic and catabolic gene was then quantified by Real-time PCR with the SYBR qPCR Master Mix (Q711-02, Vazyme, Nanjing, China) on a ABI 7500 Real-Time PCR system (Applied Biosystems, Foster City, USA). The reaction conditions were designed as follows: one cycle at 95°C for 30 s (Step 1), followed by 40 cycles at 95°C for 10 s and at 60°C for 30 s (Step 2). GAPDH was used as the normalization, and all reactions were run for three times. The primer sequences used for qRT-PCR analysis are shown in Table 1.

### 2.5. Immunohistochemical Analysis

The methods have been reported previously [13]. Briefly, intervertebral disc sections were firstly embedded in paraffin. Then, the sections were deparaffinized, and the 3% hydrogen peroxide was used to block the endogenous peroxidase. Subsequently, after antigen retrieval using pepsin (Servicebio, Wuhan, China) in 5 mM HCl, the sections were incubated with 10% goat blocking serum for 20-30 min, then with primary antibody against IL-1*β* (#12242, 1 : 100, Cell Signaling Technology, Inc. USA), p65 (#8242, 1 : 400, Cell Signaling Technology, Inc. USA), and Nrf2 (340675, Zenbio, Chengdu, China, 1 : 500) at 4°C overnight. Finally, HRP-conjugated secondary antibody (GB23302/23303, Servicebio, Wuhan, China) was added to the sections, and the sections were further counterstained using hematoxylin. Images were obtained using light microscopy (Olympus, Japan).

### 2.6. Western Blot (WB) Analysis

After different treatments, NP cells were lysed using ice-cold RIPA for 5 min, and the dissolved protein was quantified using the Protein Measurement Assay kit (PC0020, Solarbio Beijing, China). Sodium dodecyl sulphate polyacrylamide gel electrophoresis was carried out to separate the acquired proteins based on a 10% gel, and then, the separated proteins were transported onto a polyvinylidene fluoride (PVDF) membrane (EMD Millipore, Billerica, MA, USA). Blocking was carried out using nonfat milk dissolved in Tris-buffered saline-Tween (Invitrogen, San Diego, CA, USA) with the concentration of 5% for at least two hours, and the membranes were incubated with primary antibodies against: MMP-13 (820098; 54 kDa; Zenbio, Chengdu, China, 1 : 1000), type II collagen (Collagen II (1 : 1000, ab34712, Abcam), Aggrecan (ab36861, 1 *μ*g/mL, Abcam, USA), PCAN (#13110, Cell Signaling Technology, Inc. USA, 1 : 1000), Bcl2 (381702, Zenbio, Chengdu, China, 1 : 1000), Bax (200958, Zenbio, Chengdu, China, 1 : 1000), C-caspase-3 (#9664, Cell Signaling Technology, Inc. USA, 1 : 1000), MMP-3 (380816; 60 kDa; Zenbio, Chengdu, China, 1 : 1000), NOX4 (ab109225, Abcam, USA, 1 : 2000), NOX2 ab129068, Abcam, USA, 1 : 2000), SOD1(#37385, Cell Signaling Technology, Inc. USA, 1 : 1000), TNF-*α* (#3707, Cell Signaling Technology, Inc. USA, 1 : 1000), IL-6 (#12153, Cell Signaling Technology, Inc. USA, 1 : 1000), iNOS (#20609, Cell Signaling Technology, Inc. USA, 1 : 1000), COX-2 (#12282, Cell Signaling Technology, Inc. USA, 1 : 1000), NF-*κ*B p65 (D14E12, #8242, cell signaling technology, Inc., 3 Trask Lane Danvers, USA), p-p65 (Ser536; #3033, cell signaling technology, Inc., 3 Trask Lane Danvers, USA), Ik-Ba (380682, 35 kDa; Zenbio, Chengdu, China, 1 : 1,000), p-Ik-Ba (340776,35 kDa; Zenbio, Chengdu, China, 1 : 1,000), Histon H3 (Histon H3 (250182, 15 kDa; Zenbio, Chengdu, China, 1 : 1,000), and GAPDH (5174, cell signaling tecnology, Inc., 3 Trask Lane Danvers, MA 01923) at 4°C for overnight. After washed using TBST for three times, the membranes were further incubated with the secondary antibodies (380172 and 511103, Zenbio, Chengdu, China, 1 : 5,000) for another 2 hours. The target protein bands were visualized using a Tanon Imaging System (version 5200, Tanon Science & Technology Co., Ltd., Shanghai, China).

### 2.7. TUNEL Assay

After fixed by paraformaldehyde (4%) for 30 mins, the disc samples were washed by PBS for 3-4 times. Then, NP cells were permeabilized using 0.3% Triton for 5-10 mins. Next, samples were incubated with fluorescein (FITC) Tunel Cell Apoptosis Detection Kit (G1501-100T, Servicebio, Wuhan, China) for 1 hour. Nuclear was subsequently stained with DAPI solution in the dark environment. Finally, images of apoptotic cells were acquired using fluorescence microscope (Olympus, Japan).

### 2.8. Hoechst Staining

After being treated by IL-1*β* (10 ng/mL) with or without UTI (100 U/mL) for 24 h, the NP cells were incubated with 1 mL Hoechst 33342 solution for 20-30 minutes. The morphologic changes of the apoptotic cell nuclei could be detected by a fluorescence microscope (Olympus, Japan).

### 2.9. Evaluation of the Mitochondrial Membrane Potential

Mitochondrial membrane potential (MMP) could reveal the functional condition of mitochondrion. JC-1 Staining was performed via JC-1 Staining kit (Beyotime Biotechnology, Inc., Shanghai, China). Briefly, after treated with IL-1*β* (10 ng/mL) with/without UTI (100 U/mL) for 24 h, NP cells were washed by cool PBS twice and were incubated with JC-1 staining fluid in 37°C for 2 hours. Then, the NP cells were washed with JC-1 dyeing buffer solution (1X) for two times, followed by adding 2 mL of cell complete medium. Finally, a fluorescence microscope (Olympus, Japan) was used to analyze the JC-1 staining of NP cells.

### 2.10. Analysis of Apoptosis by Flow Cytometry

The method has been reported previously [14]. Annexin V-FITC Apoptosis Detection Kit (C1062M, Beyotime Biotechnology, Inc., Shanghai, China) was used. Briefly, the NP cells in different groups (Control, IL-1*β*, IL-1*β*L+UTI) were trypsinized (non-Ca^2+^), washed, and resuspended in 195 *μ*L binding buffer at a density of 5.0 − 10.0 × 10^4^ cells/mL. Next, 5 *μ*L of annexin V-FITC and 10 *μ*L of a PI solution were added to the cells for 5-10 mins in the dark environment, whereas the control group was stained with either annexin V-FITC or PI and, finally, examined via flow cytometry.

### 2.11. Enzyme-Linked Immunosorbent Assay

To obtain the conditioned media for cytokine array analysis, the NP cells were dealt with differently (Control, IL-1*β*, IL-1*β*L+UTI) for 24 without culture medium change. Then, the conditioned media were collected from each group and centrifuged (5000 rpm at 4°C for 15 minutes). The supernatants were stored at -80°C in separate until use and diluted with the appropriate standard diluents before measurement. Measurements for collagen-II, MMP-3/13, Aggrecan, MDA, SOD, TNF-*α*, IL-6, Nitrite, and PGE2 were performed on the supernatants with Enzyme-Linked Immunosorbent Assay (ELISA) kits (Westang, China). The optical density with a wavelength of 450 _OD_ was tested.

### 2.12. Analysis of Reactive Oxygen Species (ROS)

The method has been described previously [15]. After treatment, NP cells were washed for 2-3 times with PBS and then stained using FBS-free cultural medium with 2,7-dichlorodi-hydrofluorescein diacetate (DCFH-DA, 10 *μ*M, Beyotime, Shanghai, China) for 15 mins at 37°C. After that, the NP cells were once again washed for 3 times using FBS-free cultural medium. The reaction between ROS and DCFH-DA would generate dichlorofluorescein (DCFH), which could emit green fluorescence. The level of ROS was then observed by fluorescence microscope (Olympus, Japan).

### 2.13. Immunofluorescence Analysis

For aggrecan, type II collagen, MMP3, Ki-67, p65, and Nrf2 immunofluorescent staining, NP cells with different treatments were fixed using 4% PFA for 15-20 min and were permeated for another 5 min using 0.1% *v*/*v* Triton X-100. Cells were incubated with Aggrecan (GB11373, 1 : 500-1 : 1000, Servicebio, Wuhan, China), type II collagen (GB11021, 1 : 100-1 : 500, Servicebio, Wuhan, China), MMP3 (GB11131, 1 : 400-1 : 1600, Servicebio, Wuhan, China), Ki-67 (GB111141, 1 : 1200, Servicebio, Wuhan, China), NF-*κ*B p65 (D14E12, #8242, cell signaling technology, Inc., 3 Trask Lane Danvers, USA), and Nrf2 (340675, Zenbio, Chengdu, China, 1 : 100) diluted in 0.2% *w*/*v* bovine serum albumin- (BSA-) TBS for 1 h and then washed with PBS. Cells were incubated with DAPI solution (G1012-100ML, Servicebio, Wuhan, China) for visualization of nuclei and, then, incubated with FITC-conjugated goat anti-rabbit IgG (GB22303, Servicebio, Wuhan, China) and Cy3-conjugated goat anti-rabbit IgG (GB21301, Servicebio, Wuhan, China) for 30 mins in the dark environment. Fluorescence detection was performed by fluorescence microscope (Olympus, Japan).

### 2.14. Statistical Analysis

All experiments in this present study were performed for at least three times. The quantified data were presented as mean ± standard deviation (S.D). Statistical analyses were carried out using GraphPad Prism 8 (GraphPad Software Inc; La Jolla, CA) for Windows adopting one-way analysis of variance (ANOVA), followed by the Student–Neuman–Keuls post hoc test to make the comparisons between groups. *p* values less than 0.05 or otherwise indicated a statistical difference.

## 3. Result

### 3.1. Increased Expression of NF-*κ*B and Decreased Expression of Nrf2 in Degenerated Human Disc Tissue

**Figure 1:**
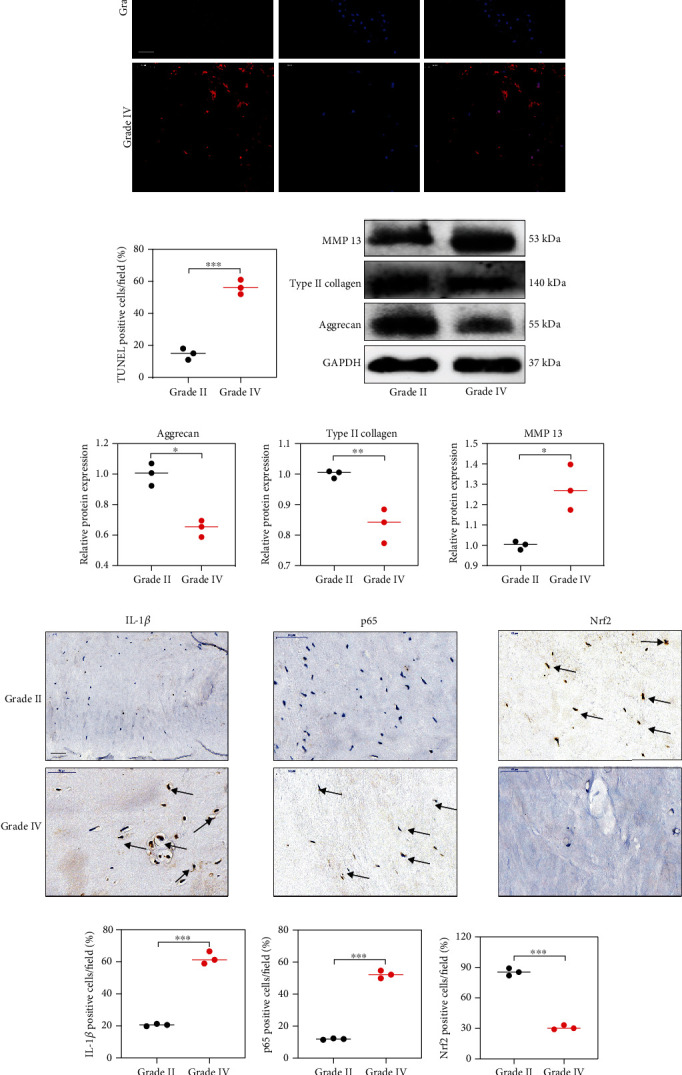
The protein expression of Nrf2 and p65 in human NP tissues. (a) The apoptotic NP cells (red) in intervertebral disc between Grade II and Grade IV were visualized via TUNEL staining and the nuclei were stained with DAPI. Scar bar = 300 *μ*m. (b) Three randomized versions were selected, and TUNEL staining-positive cells were quantified via the amount of red fluorescence. (c, d) Protein bands and quantification of expression levels of Aggrecan, Type II collagen, and MMP 13. Scar bar = 100 *μ*m. (e) Immunohistochemical results were used to examine the protein levels of IL-1*β*, p65, and Nrf2 in the human NP tissue with Grade II and Grade IV. (f) Three versions were randomly selected, and the stained cells were quantified separately. ^∗^*p* < 0.05, ^∗∗^*p* < 0.01, ^∗∗∗^*p* < 0.001, ^∗∗∗∗^*p* < 0.0001.

Intervertebral disc tissue samples were divided into nondegenerated group (grade II, *n* = 6) and degenerated group (grade IV, *n* = 6) based on the classification system [16]. As shown in Figure 1, degenerated disc tissue had higher TUNEL-positive cell (Figure 1, A). In addition, Western blot analysis also suggested that intervertebral disc tissue sample of grade IV presented with typical features of IDD, including increased expression of MMP3 and decreased expression of type II collagen and aggrecan (Figures 1(b) and 1(c)). Furthermore, immunohistochemical analysis indicated that there were significantly higher ratio of IL-1*β*-and NF-*κ*B-positive cells and lower ratio of Nrf2-positive cells in human NP tissue with degenerated intervertebral disc (Figures 1(e) and 1(f)). These results above showed that lower levels of Nrf2 may correlate with higher severity of IDD.

### 3.2. UTI Could Enhance the Cell Viability of NP Cells and Promote the Proliferative Ability of NP Cells

**Figure 2:**
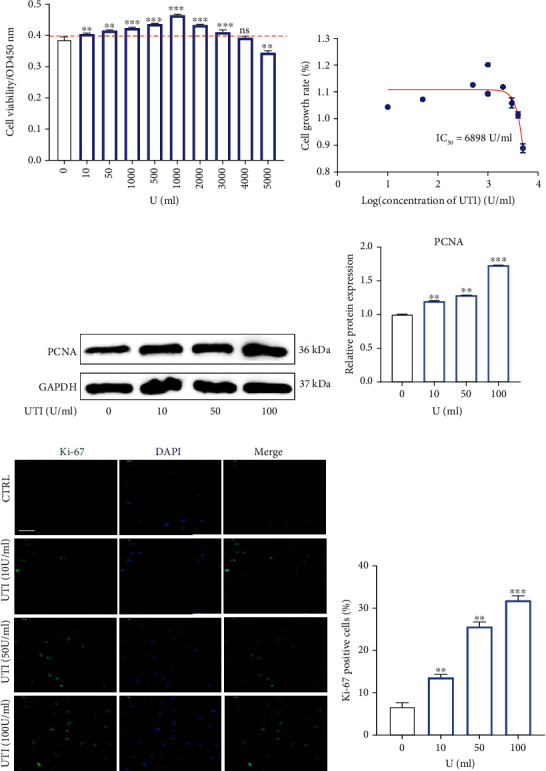
Effects of UTI on the proliferative ability in human NP cells. (a) The biological effect of UTI on the cell viability of NP cells (*n* = 5). (b) IC_50_ of UTI for human NP cells. (c, d) Protein bands and quantification of expression levels of PCNA (*n* = 3). (e, f) Expression of Ki-67 (green) was detected by the immunofluorescence. The ratios of cells with green fluorescence were calculated. Scar bar = 200 *μ*m. ^∗^*p* < 0.05, ^∗∗^*p* < 0.01, ^∗∗∗^*p* < 0.001, ^∗∗∗∗^*p* < 0.0001.

To determine the potential role of UTI on human NP cells, we firstly explored the cytotoxic effect of UTI on NP cells via a CCK-8 assay kit. As indicated in the results, UTI could significantly increase the cell viability of human NP cells without obvious cytotoxicity in vitro in a concentration below 4000 U/mL with the IC_50_ of 6898 U/mL, which suggested the safety range of UTI was wide (Figures 2(a) and 2(b)). In addition, Western blot result suggested that UTI could also promote the expression of PCNA in NP cells in a concentration-dependent manner (Figures 2(c) and 2(d)). Immunofluorescence analysis for Ki-67, a marker of cell proliferation, also confirmed the promoting effect of UTI on the proliferative ability of human NP cells (Figures 2(e) and 2(f)).

### 3.3. UTI Could Protect Human NP Cells against Apoptosis Induced by IL-1*β*

**Figure 3:**
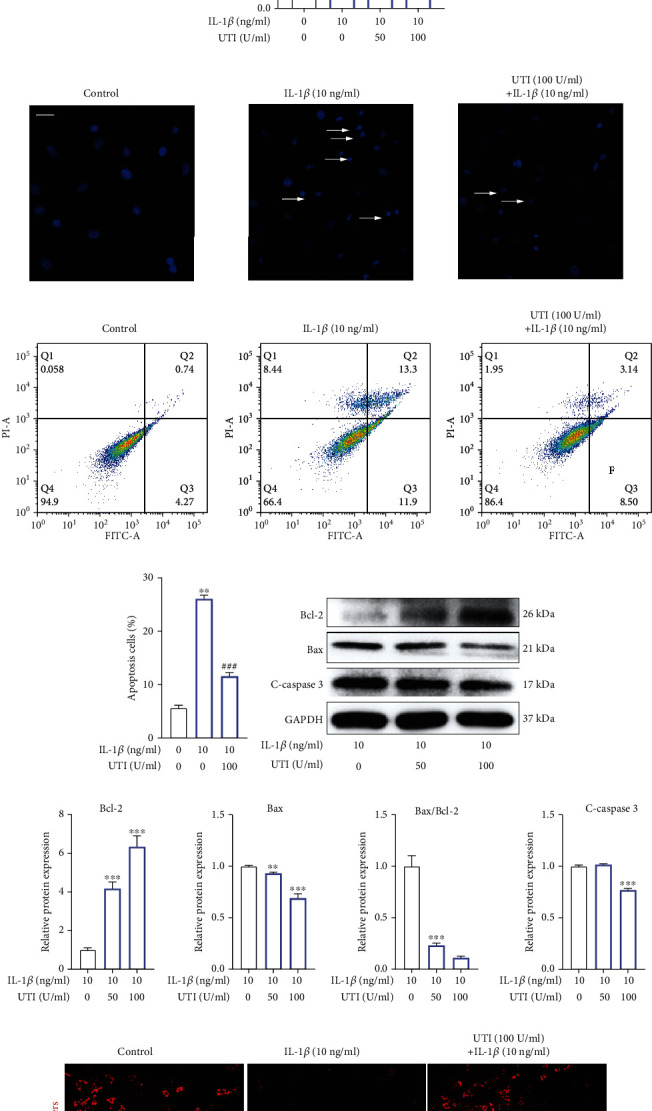
Biological effect of UTI on IL-1*β*-induced apoptosis in human NP cells. (a, b) Protein bands and quantification of expression levels of Bcl-2, Bax, and cleaved caspase 3 in IL-1*β*-induced NP cells. Bax/Bcl-2 ratios were calculated (*n* = 3). (c) Effect of UTI with indicated concentrations on cell viability of IL-1*β*-induced NP cells (*n* = 5). (d) Hoechst staining was used to conduct analysis of the nuclear morphology. Scar bar = 100 *μ*m. (e, f) The rates of apoptosis of human NP cells as determined by flow cytometry (*n* = 3). (g, h) Protein bands and quantification of expression levels of Bcl-2, Bax, and cleaved caspase 3 in IL-1*β*-induced NP cells cotreated with UTI. Bax/Bcl-2 ratios were calculated (*n* = 3). (i, j) The JC-1 monomers (red) and JC-1 aggregates (green) were detected by the fluorescent probe JC-1, and the ratios of JC-1 aggregate/monomer were calculated. Scar bar = 200 *μ*m. ^∗^*p* < 0.05, ^∗∗^*p* < 0.01, ^∗∗∗^*p* < 0.001, ^∗∗∗∗^*p* < 0.0001. ^∗^indicated the comparison between group IL-1*β* and group control. ^#^*p* < 0.05, ^##^*p* < 0.01, ^###^*p* < 0.001, ^####^*p* < 0.0001. ^#^indicated the comparison between group IL-1*β* and group UTI+IL-1*β*.

As indicated in Figure 1, the level of IL-1*β* increased significantly in degenerated intervertebral disc (Figure 1(a)), and previous studies also reported that IL-1*β* played a critical role in promoting IDD [17, 18]. Therefore, IL-1*β* was used in its present study to established human NP injury model in vitro. We pretreated NP cells with UTI and then added IL-1*β* to NP cells with for 24 h to investigate the biological effect of UTI on NP cells under IL-1*β* condition. Western blot results uncovered that IL-1*β* increased the apoptosis-related marker, Bax and cleaved-caspase 3, and suppressed the expression of antiapoptosis molecular, Bcl-2 (Figures 3(a) and 3(b)). However, this proapoptosis effect was reversed by UTI, as indicated by CCK-8 assay, Hoechst test, and flow cytometry analysis (Figures 3(c)–3(f)). In addition, western blot and analysis of MMP also confirmed the protective effect of UTI on IL-1*β*-induced human NP cells (Figures 3(g)–3(j)). Collectively, these results above showed that UTI could protect human NP cells against IL-1*β*-induced apoptosis.

### 3.4. UTI Ameliorated ECM Degradation in IL-1*β*-Induced Human NP Cells

**Figure 4:**
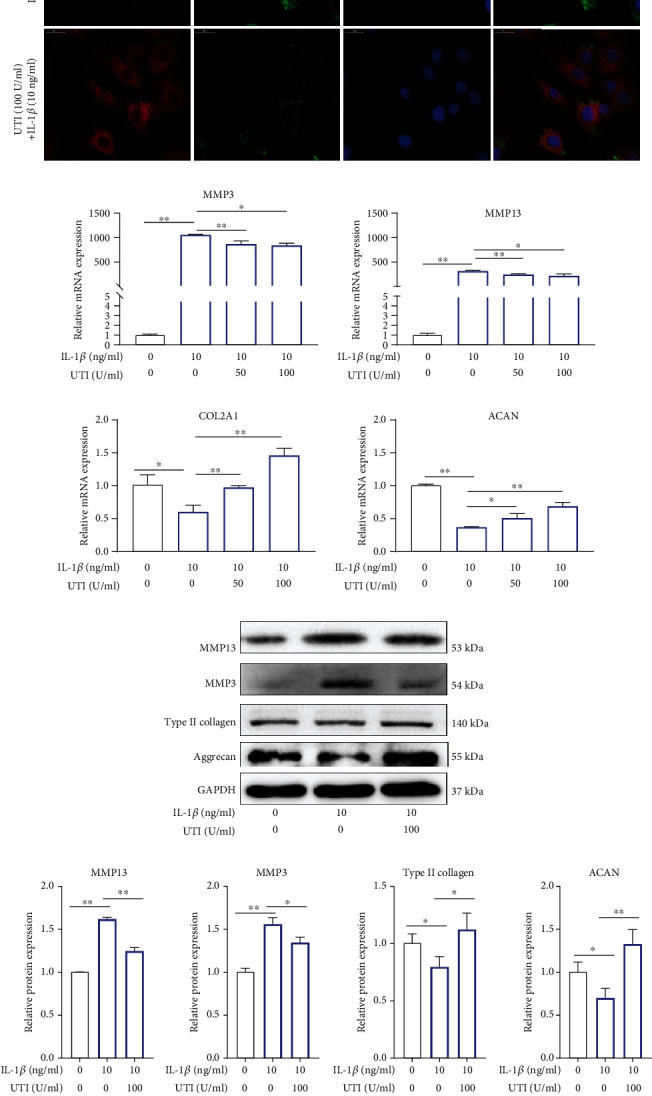
UTI ameliorated IL-1*β*-induced ECM degradation in human NP cells. (a) The expression of MMP3 and type II collagen by the immunofluorescence. Scar bar = 50 *μ*m. (b) qRT-qPCR was used to evaluate the mRNA expression of MMP 3, MMP 13, ACAN, and COL2A1 (*n* = 3). (c, d) Protein bands and quantification of protein levels of MMP 13, MMP 3, type II collagen, and aggrecan. GAPDH as an internal control (*n* = 3). (e) IL-1*β*-induced differential expression of MMP 13, MMP 3, type II collagen, and aggrecan were measured by ELISA cotreated with UTI in a dose-dependent manner in the cultural supernatant of NP cells (*n* = 3). ^∗^*p* < 0.05, ^∗∗^*p* < 0.01, ^∗∗∗^*p* < 0.001, ^∗∗∗∗^*p* < 0.0001; ^#^*p* < 0.05, ^##^*p* < 0.01, ^###^*p* < 0.001, ^####^*p* < 0.0001. The comparison among groups has been marked in figure.

The key feature of IDD is the disequilibrium of ECM synthesis and degradation [19]. To explore the protective effect of UTI on IL-1*β*-induced human NP injury, we then examined the change of ECM. As shown in Figure 4, immunofluorescence analysis for type II collagen and MMP3 suggested that IL-1*β* stimulation obviously suppressed the synthesis of type II collagen in human NP cells but increased the production of MMP3 (Figure 4(a)). Nevertheless, these abnormal alterations in the ECM of NP cells induced by IL-1*β* were significantly ameliorated by UTI (Figure 4(a)). Additionally, the results of qRT-PCR and Western blot also suggested the protective effect of UTI on IL-1*β*-induced ECM degradation in human NP cells (Figures 4(b)–4(d)).

To explore the ECM change by phenotype, in addition to immunofluorescence analysis, we also examined the secretion of catabolic proteinases in IL-1*β*-induced NP cells via ELISA, and the results revealed that pretreatment with UTI could also mitigate the increased expression of MMP3/13 induced by IL-1*β* in a concentration-dependent dose and that the protein expression of collagen type II and aggrecan were also improved by UTI, while NP cells were injured by IL-1*β* (Figure 4(e)). Conclusively, UTI ameliorated IL-1*β*-induced ECM degeneration in vitro.

### 3.5. UTI Improved Oxidative Stress Damage in Human NP Cells Induced by IL-1*β*

**Figure 5:**
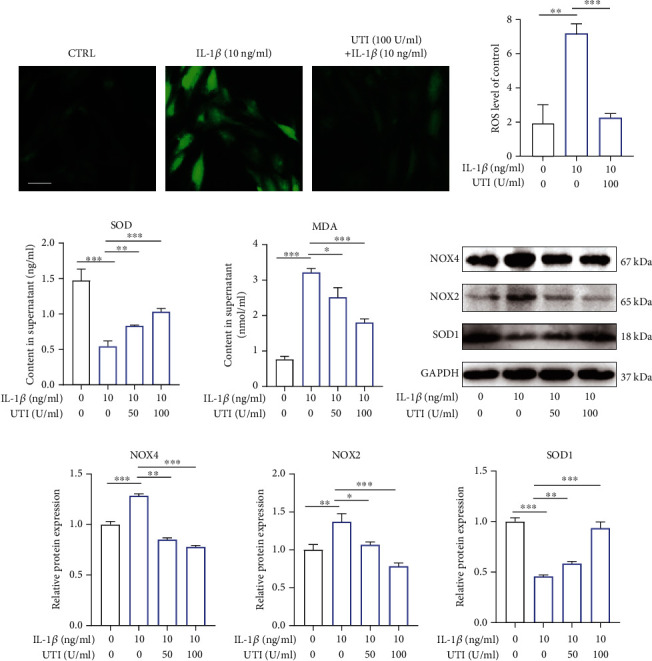
Effect of UTI on IL-1*β*-induced oxidative stress in human NP cells. (a) The distribution of ROS (green) was detected by the immunofluorescence in human NP cells treated with IL-1*β* or cotreated with IL-1*β* and UTI. Scar bar = 100 *μ*m. (b) The fluorescence strength of ROS (green) was quantified by Image J (*n* = 3). (c) IL-1*β*-induced differential levels of SOD and MDA were assessed by ELISA with UTI in a dose-dependent manner in human NP cells (*n* = 5). (d, e) Protein bands and quantification of protein levels of NOX4, NOX2, and SOD1 (*n* = 3). ^∗^*p* < 0.05, ^∗∗^*p* < 0.01, ^∗∗∗^*p* < 0.001, ^∗∗∗∗^*p* < 0.0001.

ROS content frequently indicates intracellular oxidative conditions. As shown in Figure 5, IL-1*β* increased the ROS level by more than 3-fold in NP cells (Figures 5(a) and 5(b)). However, after pretreatment with UTI, the increased ROS level induced by IL-1*β* was ameliorated (Figures 5(a) and 5(b)). ELISA analysis also suggested the suppressive effect of UTI on IL-1*β*-induced oxidative stress (Figure 5(c)). At the level of molecule, Western blot analysis suggested that prooxidative stress-related proteins, Nox2/4, were significantly increased by IL-1*β* and that the expression of antioxidative stress-related protein, SOD1, was inhibited. Nevertheless, these effects were all reversed by UTI in a dose-dependent manner (Figures 5(d) and 5(e)).

### 3.6. UTI Exerted Anti-Inflammatory Effect via Regulated Inflammation-Related Mediators in Human NP Cells Induced by IL-1*β*

**Figure 6:**
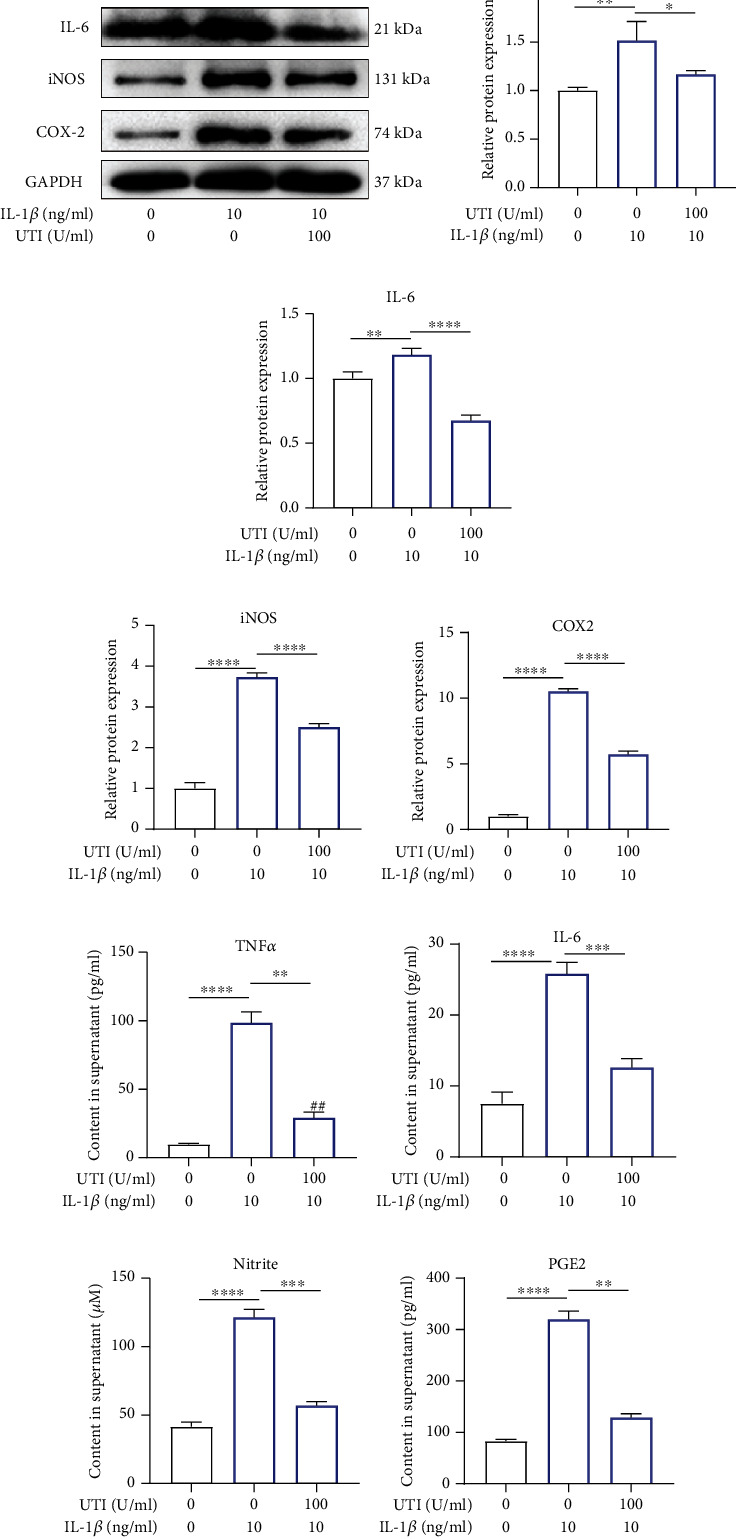
Impact of UTI on IL-1*β*-induced expression of inflammatory factors in human NP cells. (a–e) Protein bands and quantification of protein expression of TNF-*α*, iNOS, IL-6, and COX-2. GAPDH as an internal control (*n* − 3). (f–i) Differential expression of TNF-*α*, IL-6, Nitrite, and PGE2 were quantified by ELISA assay (*n* = 5). ^∗^*p* < 0.05, ^∗∗^*p* < 0.01, ^∗∗∗^*p* < 0.001, ^∗∗∗∗^*p* < 0.0001.

During IDD, excessive focal inflammatory response will damage the normal biological function of NP cells in a positive-feedback manner, inhibiting the abnormal inflammatory microenvironment in disc tissue becomes a therapeutic target [20, 21]. Therefore, we further explored the impact of UTI on IL-1*β*-induced inflammatory response. COX-2 and iNOS have been previously reported to be the two key inflammatory mediators during IDD. In this present study, we firstly examined the protein level of COX2 and iNOS in NP cells treated by L-1*β* with or without UTI. As shown in Figure 6, UTI dramatically suppressed IL-1*β*-induced expression of COX2 and iNOS as well as proinflammatory factors, IL-6 and TNF-*α* (Figures 6(a)–6(e)). In addition, The generation of endogenous of IL-6 and TNF-*α* during IL-1*β* stimulation was also inhibited by UTI (Figures 6(f) and 6(g)). ELISA also demonstrated that UTI ameliorated the production of endogenous NO and PGE2 (Figures 6(h) and 6(i)).

These results indicated that UTI inhibited the IL-1*β*-induced generation of inflammatory mediators and cytokines.

### 3.7. UTI Regulated IL-1*β*-Induced NF-*κ*B Activation in NP Cells

**Figure 7:**
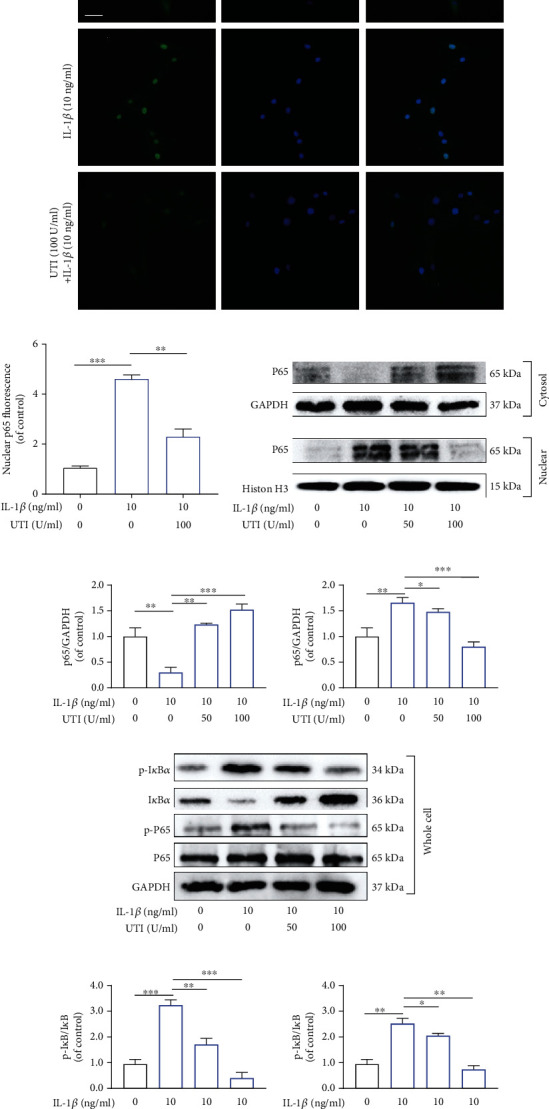
Effects of UTI on the activation of NF-*κ*B pathway induced by IL-1*β* in human NP cells. (a, b) The nuclei translocation of p65 in different groups was visualized. Intensity of nuclear p65 fluorescence was quantified (*n* = 3). Scar bar = 200 *μ*m. (c, d) Differential expressions of p65 in cytoplasm and in nucleus were visualized and quantified by WB, respectively (*n* = 3). (e, f) Protein levels of I*κ*B*α*, p65, and their phosphorylated forms were analyzed by WB in different groups (*n* = 3). ^∗^*p* < 0.05, ^∗∗^*p* < 0.01, ^∗∗∗^*p* < 0.001, ^∗∗∗∗^*p* < 0.0001.

NF-*κ*B pathway has been widely reported to be involved in the generation of inflammation response and oxidative stress during IDD [22–24]. As shown in Figure 7, without IL-1*β* intervention, p65 was primarily distributed in the cytoplasm of NP cells, whereas the amount of p65 was elevated dramatically in the nuclei and was reduced in the cytoplasm following IL-1*β* treatment. This phenomenon suggested that IL-1*β* treatment dramatically increased the nuclear translocation of p65. However, this effect was suppressed by UTI (Figures 7(a) and 7(b)). Additionally, Western blot analysis also confirmed the suppressive effect of UTI on IL-1*β*-triggered nuclear translocation of p65 (Figures 7(c) and 7(d)). In general, NF-*κ*B exists as an inactive condition in the cytoplasm due to its interaction with I*κ*B family proteins which suppress the nuclear translocation of NF-*κ*B. Once stimulated, the phosphorylated IKK-*β* activates I*κ*B-*α* which becomes phosphorylated and ensuing degraded, and thus, NF-*κ*B will be released and translocate into cell nucleus [25]. Therefore, we also explored the change of I*κ*B-*α* and found that IL-1*β* intervention markedly promoted the phosphorylation and degradation of I*κ*B*α* in the cytoplasm. The increased amount of p65 in the cytoplasm of the NP cells treated by UTI also confirmed the suppression of IL-1*β*-activated nuclear translocation of p65 (Figures 7(e) and 7(f)).

In conclusion, UTI inhibited the IL-1*β*-induced activation of NF-*κ*B by inhibiting I*κ*B*α* phosphorylation in the cytoplasm and thus suppressed the amount of p65 translocation into the nuclei of the NP cells.

### 3.8. UTI Regulated IL-1*β*-Induced Apoptosis, Inflammation Response, and Oxidative Stress via Nrf2/NF-*κ*B Signaling Pathways in Human NP Cells

**Figure 8:**
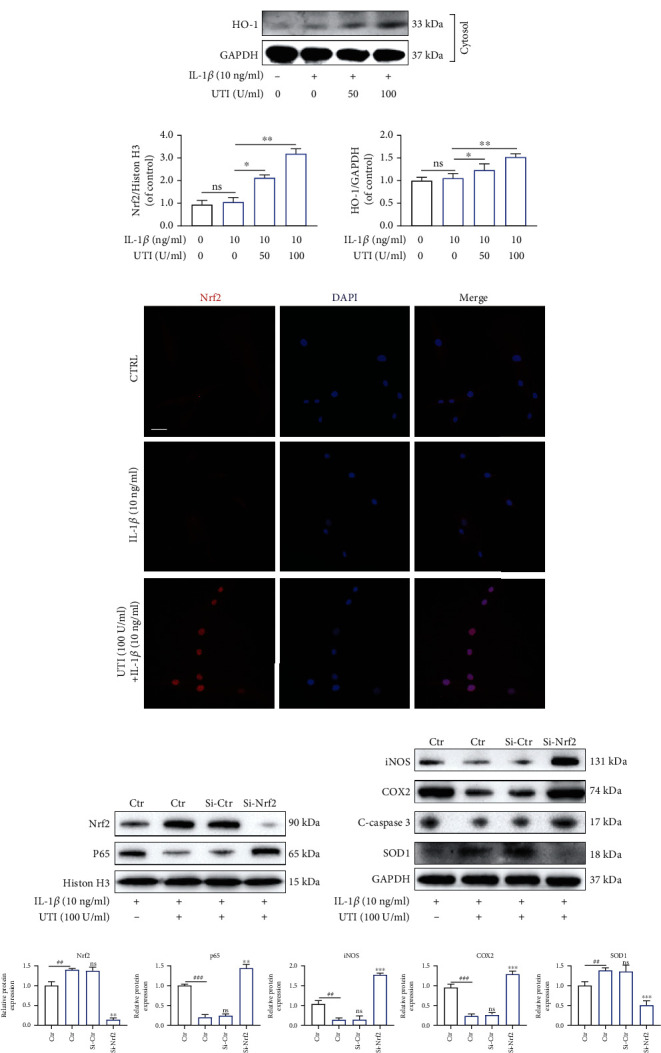
Effects of UTI on Nrf2/HO-1 pathway in human NP cells induced by IL-1*β*. (a, b) Expression at protein level of Nrf2 in nucleus and HO-1 in cytoplasm were analyzed and quantified (*n* = 3). (c) The nuclear translocation of Nrf2 was visualized via immunofluorescence. Scar bar = 200 *μ*m. (d, e) After knockdown of Nrf2, the protein amount of Nrf2 and p65 in nucleus and iNOS, COX2, c-caspase3, and SOD1 in cytoplasm in NP cells were presented by WB (*n* = 3). (f) Quantification of WB results of Nrf2, p65, iNOS, COX2, c-caspase3, and SOD1. ^∗^*p* < 0.05, ^∗∗^*p* < 0.01, ^∗∗∗^*p* < 0.001, ^∗∗∗∗^*p* < 0.0001.

As presented, Western blot results suggested that IL-1*β* suppressed the amount of Nrf2 in the nucleus and HO-1 in cytoplasm of human NP cells, whereas these effects were ameliorated by UTI (Figures 8(a) and 8(b)). Notably, no obvious difference was observed between normal cultured NP cells and IL-1*β*-treated NP cells in terms of the expression level of Nrf2 and HO-1, although NP cells with IL-1*β* seem to express higher level of these two proteins (Figures 8(a) and 8(b)). Furthermore, immunofluorescence analysis for Nrf2 demonstrated that UTI enhanced the nuclear translocation of Nrf2, which was consistent with the results of WB (Figure 8(c)).

To further explore the potential association between Nrf2 and pathological features of human NP cells induced by IL-1*β*, we used small interfering RNA for Nrf2 (siRNA-Nrf2) to establish the knockdown model of Nrf2 in NP cells. As shown in Figure 8, siRNA-Nrf2 evidently inhibited the expression of Nrf2, suggesting the successful establishment of Nrf2 knockdown (Figure 8(d)). However, following knockdown of Nrf2, the amount of p65 in nucleus of NP cells was negatively increased during stimulation by IL-1*β* with or without UTI (Figure 8(d)). What is more, the expression of SOD1 was decreased, whereas the expression of iNOS, COX2, and c-caspase3 was increased after pretransfection with siRNA-Nrf2 (Figures 8(e) and 8(f)). Taken together, the Nrf2 pathway was involved in UTI-mediated NF-*κ*B signaling suppression, and subsequent regulation of apoptosis, inflammation response, and oxidative stress in human NP cells treated with Il-1*β*.

## 4. Discussion

In the context of the fourth industrial revolution, the proportion of static work in human labor is increasing, and the spine is under an unprecedented state of continuous fatigue. According to the epidemiological survey, more than 80% of the population worldwide would suffer from LBP in their lifetime [3]. As a major cause of LBP, IDD has been a multifactorial disease with typical features of ECM degradation and an increasing reduction in the number of NP cells. Thus, delaying the ECM degradation and reducing the cell apoptosis become the critical aims in the treatment of IDD.

An increasing number of studies have suggested excessive inflammatory response and oxidative stress may be the major causes of IDD [26]. Accumulating the production of local inflammatory mediators, such as IL-1*β*, could trigger NP cell injury [27]. In addition, injured NP cells could further produce proinflammatory and oxidative stress-related cytokines which further exacerbate the progression of IDD via a malignant positive feedback loop [28]. Therefore, genetic or pharmacologic interventions targeting at the inflammatory mechanisms and ECM degradation may be a novel strategy for treating IDD. IL-1*β* is reported to correlate closely with multiple pathological process of IDD [29]. Therefore, in this present study, we established IDD model in vitro using IL-1*β*, and the results suggested the protective role of UTI in IL-1*β*-induced cell apoptosis, inflammation response, oxidative stress, and ECM degradation via Nrf2/NF-*κ*B pathway in human NP cells.

One of the major characteristics of IDD is decreasing number of NP cells [29]. Accumulating studies have shown that cell apoptosis played a vital role in reducing NP cells [30, 31]. Firstly, we evaluated the biological effect of UTI on the proliferative ability of NP cells induced by IL-1*β*. As demonstrated in the results, UTI elevated the cell viability and proliferative ability of NP cells. On the other hand, we also explored the effect of UTI on NP cell apoptosis. Apoptosis is mainly triggered by two classical pathways: death receptors pathway and mitochondria pathway. In fact, the intrinsic pathway is frequently coregulated by Bcl-2 and Bax [32]. The ratio of Bax/Bcl-2 can be used to estimate level of apoptosis. As indicated by the results, IL-1*β* aggravated the apoptosis of NP cells, whereas this damaging effect was improved by UTI. Further results of CCK-8 assay, Hoechst test, flow cytometry analysis, and analysis of MMP also confirmed the protective effect of UTI on NP cells. Taken together, the results above suggested that UTI has proproliferative and antiapoptotic effects on NP cells stimulated by IL-1*β* and that the antiapoptotic effect may, at least partly, be attributed to the suppression of the mitochondrial apoptosis pathway.

During the process of IDD, increasing MMPs, and decreasing type II collagen and aggrecan can be another two typical features of degenerated disc. Under normal condition, the type II collagen and aggrecan constitute the highly hydrated nature of the NP, which can help keep disc height [33]. However, abnormal generation of matrix-degrading enzymes (MMPs and ADAMTs) due to various factors will result in ECM degradation. In addition, excessive accumulation of inflammatory mediators can further increase the expression of MMPs and ADAMTs, accelerating the progression of IDD [34]. Following, we stimulated NP cells with IL-1*β*, and the NP cells showed increased expression of MMP3/13 and decreased expression of type II collagen and aggrecan. Nevertheless, administration of UTI suppressed these abnormal changes, indicating the suppressive effect of UTI on IL-1*β*-induced ECM degradation.

Abnormally, increasing proinflammatory mediators, such as IL-6, can also accelerate IDD [34, 35]. We also assessed the potential effect of UIT on the IL-1*β*-induced inflammatory response and found that the increased levels of IL-6 and TNF-*α* induced by IL-1*β* were inhibited by UTI, as demonstrated by Western blot analysis and ELISA results. In addition, the proinflammatory mediators, iNOS and COX-2, were also negatively regulated by UTI. On the other hand, overproduction of ROS due to environmental stress or inflammation was also a critical factor contributing to IDD [36]. In our study, IL-1*β* caused increased levels of ROS, MDA, and NOX2/4 but decreased level of SOD, whereas UTI mitigated these changes. Collectively, UTI showed a protective effect on IL-1*β*-treated NP cells by suppressing the excessive activation of inflammatory responses and oxidative stress.

Increasing studies have proved that activated NF-*κ*B pathway is associated with the progression of IDD and, widely, involved in the proapoptotic effect, inflammatory response, oxidative stress, and the production of ECM-degradation proteinases [37–39]. IL-1*β* stimulation can activate I*κ*B*α*, which subsequently facilitates the nuclear translocation of p65. The activated NF-*κ*B pathway will further promote the generation of proinflammatory molecules (COX-2 and iNOS), which could result in the ECM degradation [18, 40]. Suppressing the activation of NF-*κ*B pathway has been considered as a promising therapeutic insight against IDD. A recent study by Yi et al. revealed that inhibiting NF-*κ*B pathway could decrease apoptosis and inflammation response in injured NP cells [22]. In this present study, we found that UTI could suppress the nuclear translocation of p65 and reduce the cell apoptosis and abnormal generation of inflammatory mediators, cytokines, and MMPs. Although not all of those effects of p65 are direct, UTI, at least partly, protects NP cells against apoptosis and the inflammatory response by suppressing NF-*κ*B pathway.

According to this current study, oxidative stress injury frequently occurs with inflammatory response during IDD. The results above also suggested the inhibitory effect of UTI on the level of oxidative stress. Previous studies have revealed that Nrf2/HO-1 pathway plays a key role in anti-inflammation, antioxidation, and reducing mitochondrial damage [41, 42]. Tang et al. ever reported that activating Nrf2 could suppress the NF-*κ*B signaling pathway and ameliorate the progression of osteoarthritis (OA) [43]. Dawei song found that theophylline could activate Keap1/Nrf2/ARE pathway to exert effects of antioxidation and slow down the IDD [44]. Cuadrado et al. also revealed that the deficiency of Nrf2 can lead to the enhancement of NF-*κ*B activity and cytokine production [45]. However, whether Nrf2/HO-1 pathway is also associated with the protective effect of UTI on NP cells remains elusive. In this present study, we also found the decreasing expression of Nrf2 in degenerated disc tissue. In addition, we used siRNA-Nrf2 to knock down the expression of Nrf2, and the results demonstrated that suppressed Nrf2 aggravated the cell apoptosis, oxidative stress, and inflammation reactions. Interestingly, the suppression of Nrf2 also led to the activation of NF-*κ*B pathway. Taken together, UTI may exert antiapoptotic, anti-inflammatory, and antioxidative function via activating the Nrf2/HO-1 axis and suppressing the NF-*κ*B pathway in IL-1*β*-treated human NP cells.

In conclusion, this current study uncovered that UTI could protect human NP cells against IL-1*β* stimulation by inhibiting apoptosis, inflammatory response, oxidative stress, and ECM degradation in human NP cells. In addition, the protective effect of UTI on IL-1*β*-treated NP cells was accomplished by activating the Nrf2/HO-1 signaling axis and suppressing NF-*κ*B signaling pathway. Therefore, UTI may be potential therapeutic medicine to delay the progression of IDD. However, further work needs to be done, such as in vivo experiment, before it can be known whether UTI is efficacious as a reliable treatment for IDD.

## Figures and Tables

**Table 1:** Sequence of primers for qRT-PCR.

Gene name		Sequence 5′ to 3′
MMP-3	F	GATGCGCAAGCCCAGGTGTG
	R	GCCAATTTCATGAGCAGCAACGA
MMP13	F	TCAGGAAACCAGGTCTGGAG
	R	T G A C G C G A A C A ATA C G G T TA
Collagen type II	F	AATTCCGACCTCGTCATCAG
	R	GCCTGGATAACCTCTGTG
Aggrecan	F	TGAGCGGCAGCACTTTGAC
	R	TGAGTACAGGAGGCTTGAGG
GAPDH	F	GCCGCTTCTTCTCGTGCAG
	R	AT G G AT C AT T G AT G G C G A C A A C AT

## Data Availability

Data is available on request.

## Supplementary Materials

Supplementary Materials The human NP cell phenotype was confirmed by identifying the expression of type II collagen and aggrecan. Immunofluorescence analysis for collagen type II and toluidine blue staining for aggrecan were used in this present study. The results were shown in Supplementary Figure 1. Supplementary Figure 1: culture and identification of human nucleus pulposus (NP) cells. (a) The human NP cells of passage 1 and 4 under light microscope. Scar bar = 400 *μ*m. (b) Immunofluorescence analysis for collagen type II of NP cells of passage 1 and 4. (c) Toluidine blue staining for aggrecan of NP cells of passage 1 and 4. Scar bar = 200 *μ*m.
