# Supplementary material for: Ulinastatin Ameliorates IL-1β-Induced Cell Dysfunction in Human Nucleus Pulposus Cells via Nrf2/NF-κB Pathway
Source: Oxid Med Cell Longev. 2021 Apr 21;2021:5558687. doi: 10.1155/2021/5558687 (PMC8084647; doi:10.1155/2021/5558687)
Supplement: Supplementary Materials — The human NP cell phenotype was confirmed by identifying the expression of type II collagen and aggrecan. Immunofluorescence analysis for collagen type II and toluidine blue staining for aggrecan were used in this present study. The results were shown in Supplementary Figure 1. Supplementary Figure 1: culture and identification of human nucleus pulposus (NP) cells. (a) The human NP cells of passage 1 and 4 under light microscope. Scar bar = 400 μm. (b) Immunofluorescence analysis for collagen type II of NP cells of passage 1 and 4. (c) Toluidine blue staining for aggrecan of NP cells of passage 1 and 4. Scar bar = 200 μm. [file 5558687.f1.docx]

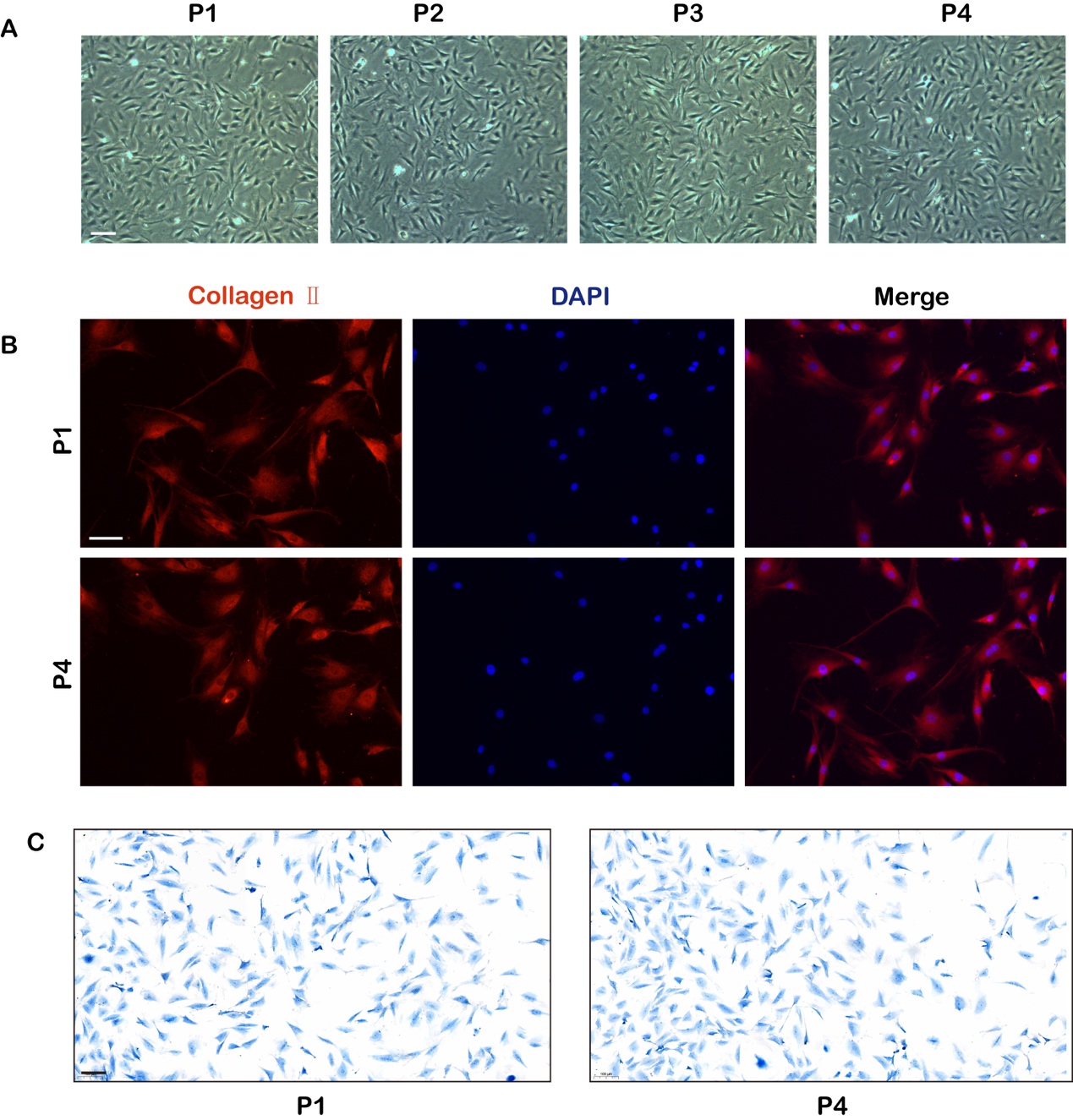


**Supplementary Figure 1 Culture and identification of human nucleus pulposus (NP) cells.** (A) The NP cells of passage 1 and 4 under light microscope; Scar bar=400um.(B) Immunofluorescence analysis for collagen type II of NP cells of passage 1 and 4; (C) Toluidine blue staining for aggrecan of NP cells of passage 1 and 4. Scar bar=200um.
